# Role of Clay Minerals in Natural Media Self-Regeneration from Organic Pollution-Prospects for Nature-Inspired Water Treatments

**DOI:** 10.3390/molecules29215108

**Published:** 2024-10-29

**Authors:** Abdelkrim Azzouz, David Dewez, Amina Benghaffour, Robert Hausler, René Roy

**Affiliations:** 1 Nanoqam, Department of Chemistry, Université du Québec à Montréal, Montreal, QC H3C 3P8, Canada; dewez.david@uqam.ca (D.D.); amina.benghaffour@uqam.ca (A.B.); roy.rene@uqam.ca (R.R.); 2 Department of Construction Engineering, École de Technologie Supérieure, Montreal, QC H3C 1K3, Canada; robert.hausler@etsmtl.ca; 3 Glycosciences and Nanomaterials Laboratory, Université du Québec à Montréal, Montreal, QC H3C 3P8, Canada

**Keywords:** organic pollutants, clay minerals, soils, aquatic media, remediation, oxidative processes

## Abstract

Pollution from organic molecules is a major environmental issue that needs to be addressed because of the negative impacts of both the harmfulness of the molecule structures and the toxicity that can spread through natural media. This is mainly due to their unavoidable partial oxidation under exposure to air and solar radiation into diverse derivatives. Even when insoluble, the latter can be dispersed in aqueous media through solvatation and/or complexation with soluble species. Coagulation–flocculation, biological water treatments or adsorption on solids cannot result in a total elimination of organic pollutants. Chemical degradation by chlorine and/or oxygen-based oxidizing agents is not a viable approach due to incomplete mineralization into carbon dioxide and other oxides. A more judicious strategy resides in mimicking natural oxidation under ambient conditions. Soils and aqueous clay suspensions are known to display adsorptive and catalytic properties, and slow and complete self-regeneration can be achieved in an optimum time frame with a much slower pollution throughput. A deep knowledge of the behavior of aluminosilicates and of oxidizing species in soils and aquatic media allows us to gain an understanding of their roles in natural oxidative processes. Their individual and combined contributions will be discussed in the present critical analysis of the reported literature.

## 1. Introduction

**Scheme 1:**
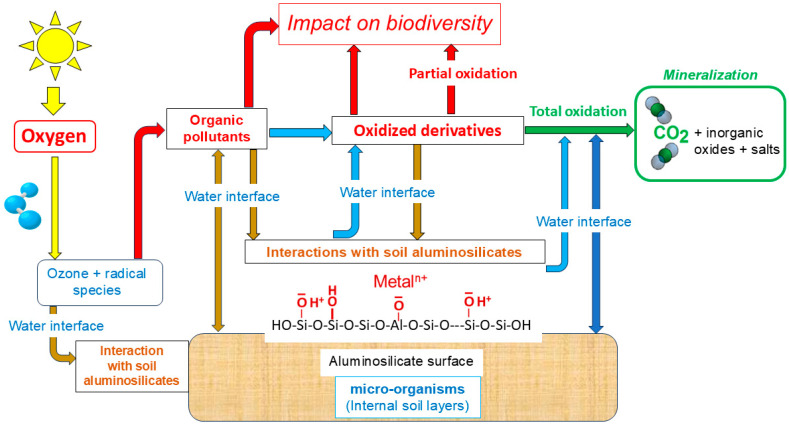
Factors and impacts of partial oxidative degradation of organic pollutants on biodiversity. The arrow colors are defined by the species and/or process step marked by the same color.

Synthetic organic molecules are major environmental pollutants because of their low and slow degradability in soils and aqueous media upon exposure to oxidation-reduction processes [1]. This unavoidably generates harmful intermediates that negatively impact biodiversity and subsequently human health through complex physical–chemical, biochemical and often interdependent processes. The presence of minerals in natural media exposed to air and solar radiation should spontaneously favor the non-bacterial oxidation of organic molecules at the expense of the other processes, even leading to total mineralization into harmless oxides during longer exposures (Scheme 1).

Due to the lack of upstream treatments of wastewater and gas emissions with strategies that take into account pollutant chemistry and interactions with the natural host media, all remediation attempts so far were doomed to fail. A deep knowledge of the natural capacity of such host media to decompose organic pollutants is an essential requirement for any further remediation approaches, and nature-inspired approaches are probably the most promising for such a purpose.

**Scheme 2:**
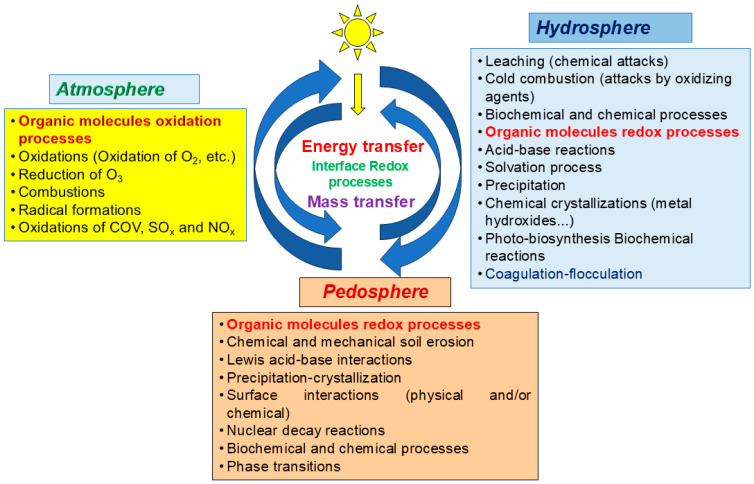
Interdependence of natural physico-chemical processes via mass and energy transfer.

For instance, the atmosphere is often polluted by oxides of carbon (COx such as CO and CO_2_), sulfur (SOx) and nitrogen (NOx) along with volatile organic compounds (VOCs). These gas pollutants are recognized as being the main causes of the greenhouse effect, SMOG, acid rain, ozone layer depletion and other phenomena at their interfaces with other media. The reciprocal interactions of the different parts of the environment (atmosphere, hydrosphere and pedosphere) are mainly due to their interdependence via mass and energy transfer (Scheme 2).

This explains why a specific pollution, for instance, in water bodies, unavoidably results in direct negative impacts on the quality of not only the atmosphere but also of soils, glaciers and aquatic media (oceans, seas, lakes, rivers, streams, wetlands and ground water). For gas pollutants, this issue may be tackled by reducing the flue emissions throughput or at least through effective gas capture technologies coupled with consecutive pollutant conversion into energy or added-values products to reduce the effect of mass/energy transfer to other parts of the environment.

## 2. Need for a Global Vision of Organic Pollution

**Scheme 3:**
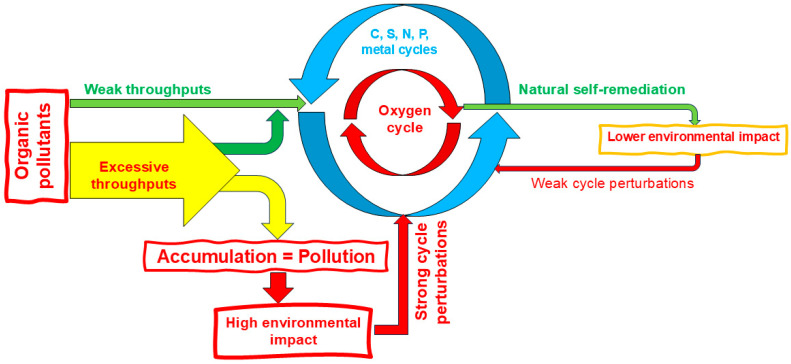
Effect of excessive pollution throughput of organic molecules on natural cycles’ equilibria. The arrow colors are defined by the species and/or process step marked by the same color.

Organic compounds are chemical species that are not necessarily included in the metabolisms of flora and fauna; more specifically, they often originate from synthetic procedures. They can even be alien compounds with respect to the natural cycles of purely inorganic media, and their mere occurrence is a perturbation of the global environmental equilibrium. Even if they are biodegradable, as is the case for most recent forms of agro-industrial waste, their production throughput often surpasses the self-regeneration capacity of the natural host media, leading to the accumulation of waste and pollution (Scheme 3).

A small environmental impact still persists even in the case of the total metabolization of the alien organic molecules, inasmuch as increases in the perturbation of the global environmental equilibrium and entropy are unavoidable. As long as there is no global vision of pollution that includes both mass and energy transfers, the global thermodynamic equilibrium remains disturbed with potential impacts on surrounding media, even if these are barely perceptible.

Water and aquatic media also contain diverse organic pollutants which are produced by human activities (Table 1). This includes pesticides, drugs, polycyclic aromatic hydrocarbons (PAHs), polychlorinated biphenyls (PCBs), organic dyestuffs [2], brominated diphenyl ethers, polyaromatic hydrocarbons (PAH), dioxins, solvents, hydrocarbons [2,3,4,5] and many others.

Depending on their solubility in water, organic pollutants present in soils could be leached into streams, rivers and seas by runoff waters or, conversely, retained by soils displaying high hydrophobicity. Some of these pollutants already exhibit an intrinsic toxicity, and their partial decomposition often induces additional toxicity by producing harmful derivatives leading to human health issues [43]. Consequently, regardless of their occurrence in a given natural host medium, they can easily be transferred to another one, thereby inducing an even more pronounced spread of ecotoxicity in the environment.

Other organic pollutants such as drugs, antibiotics, hormone regulators, estrogens and others have already been found to affect human health and biodiversity [44,45,46,47]. Drugs used in human medicine including analgesics and anti-inflammatories such as Acetaminophen, ibuprofen, Naproxen (Anaprox), Aspirin, Diclofenac, hormone regulators (17-β-estradiol, estriol, estrone, testosterone, 17-α-Ethinylestradiol) and antibiotics (Chlortetracycline, Tylosin, Erythromycin, Sulfamethoxazole, Tetracycline, etc.) turned out to be fairly harmful towards biodiversity even in trace amounts [48]. Among these, 17-a-ethynylestradiol had already been reported to cause male feminization in aquatic populations [49,50], an enhancement of bacteria immunity, and rise in resistant bacteria [51,52,53]. Waters could contain other diverse organic species that may even trigger cardiovascular and neurological disorders and diseases. In these cases, primary and secondary water treatments are not sufficient, and should be complemented by additional steps targeting the total mineralization of both the organic pollutants and their intermediates. Under optimal conditions, the catalytic oxidation of organic pollutants containing nitrogen, sulfur or other hetero atoms is expected to generate carbon dioxide (CO_2_), oxides of sulfur, nitrogen and phosphorus, sulfate and nitrate anions [2,54].

## 3. Conventional Techniques for Organic Pollutant Elimination

The prevention and/or remediation of soil pollution are quite difficult to achieve and depend on the nature and concentration of the pollutants and the extent and depth of the contaminated surface. Organic pollutants are expected to be largely retained in soils through hydrophobic interactions with metal oxides’ surfaces, particularly with silica. When concentrated within the outermost soil layers, remediation may be achieved through either in situ oxidative processes under prolonged exposure to air and solar radiation, spreading micro-organism suspension or thermodegradation through combustion with provoked surface fires. Deep soil contamination imposes a multi-step extraction–treatment of the polluted layer. This requires temporary industrial devices for the excavation of the earth to be treated (optional depending on the methods), a percolation process or gas injection and leachate recovery and treatment before returning the treated soil to its original place.

Most organic molecules are already functionalized, while others can be functionalized by partial natural oxidation. Further pH fluctuations may promote their polarity and solubility in stream waters. In this situation, the organic pollutants are leached and recovered in drained waters to be treated. As a result, soil remediation will only be achieved via water treatments, after connecting the drained water stream to treatment facilities. This is why water treatments play a key role in soil remediation and why partial natural oxidation of the organic molecules is a key step that depends on the soil type and chemical composition, more specifically its clay content [18,19,20,24].

Water can be treated through physical or chemical/biochemical techniques. Conventional primary and secondary water treatments often result in an incomplete elimination of the organic pollutants [55]. In such treatments, precipitation, coagulation, flocculation, aeration and biological techniques are classical steps, but they have a low efficiency in organic pollutant removal. The treated water contains residual amounts of organic compounds that lead to their bioaccumulation. Their unavoidable oxidation upon exposure to air and sun radiation results in oxidized derivatives that exhibit a lower biodegradability compared to the parent molecules [56,57] and have long-term negative impacts on biodiversity and human health once released into nature [58]. Synthetic organic species already display an intrinsic toxicity when they are chemically stable, but their partial oxidation constitutes an additional source of toxicity, by generating diverse harmful intermediates according to their molecular structure and chemical stability. This stability was found to be directly proportional to their oxidation levels [2,59,60]. In other words, acidic species are much more chemically stable compared to their corresponding ketone and alcohol intermediates. This explains the persistence of some acidic species, which are fairly refractory to conventional oxidizing agents even under sun exposure, displaying a weak capacity to be “metabolized” by natural host media or by micro-organisms in biological water treatments.

Organic pollutant removal through physical techniques involves no change to chemical structures but only their separation from liquids by filtration, coagulation–flocculation, solvent sublation and/or aeration. Nanofiltration and reverse osmosis are also physical techniques for the elimination of micro-pollutants from stream water [61]. All these of these methods are intended for the treatment of highly polluted waters in spite of some major shortcomings. The latter consists mainly of a limited liquid–liquid interface or liquid–gas transfer of the organic molecules, the potential use of organic solvents imposed by the persistence of residual trace amounts and the production of undesired sludges by filtration and coagulation–flocculation that must be handled as additional pollutants. In addition, the use of coagulating agents containing aluminum or iron is often subject to controversy, particularly when targeting drinking water [62]. Free aluminum and iron have already been reported to favor Alzheimer’s disease [63,64] and Parkinson’s symptoms, respectively [65]. Possible solutions in this regard involve the use of harmless and biorecyclable aluminosilicate-based coagulating agents [62,66].

Chemical water treatments are intended to decompose the organic molecules into less harmful/harmless derivatives or to induce structural changes for their separation and elimination. Among these, chlorination, saponification–extraction and oxidation processes are particularly efficient. One of the most commonly employed methods for water disinfection is undoubtedly chlorination, which is employed against bacteria and viruses. The use of chlorine generates non-desired disinfection by-products (DBPs), i.e., chlorinated derivatives that display toxicity towards human health and aquatic biodiversity [67]; some of these, such as trihalomethanes (THMs), can lead to cancers in the digestive tract and genito-urinary system [68]. The use of other oxidizing agents such as hydrogen peroxide, oxygen under light radiation, ozonation and others in lieu of chlorine could be a promising alternative, but the elimination of persisting traces of oxidized intermediates needs further improvements. Further investigations are still in progress in many research laboratories.

Biological remediation processes appear to be green routes for water treatment. Nevertheless, the use of such methods remains limited by major drawbacks related to unavoidable fluctuations in pH and bacterial activity and potential contaminations with other micro-organisms and/or diverse chemicals. Large lagoon pools and significant investment costs for preserving soil viability are additional constraints that restrict biological remediation techniques to, at most, primary or secondary water treatment rather than for the total removal of organic pollutants.

## 4. New Strategies for Thorough Remediation of Polluted Media

Notwithstanding the fact that different approaches have been undertaken to tackle the organic pollutant issues [2], technical and economic reasons have been major obstacles to adapting specific treatment strategies to wastewater arising from different sources. For instance, total mineralization into CO_2_, water and relatively less harmful SOx and NOx may be achieved through more or less intense oxidative remediation processes adapted according to the chemical stability of each type of organic pollutant. Photo-oxidation (O_2_/UV), Fenton process (H_2_O_2_/Fe^2+^), Wet Air Oxidation (WAO) and the use of ozone (O_3_) are probably the most common methods for the elimination of organic molecules and bacteria [69]. Nonetheless, such processes are much less effective for achieving total mineralization of the organic pollutants as compared to their combinations designated as Advanced Oxidation Processes or AOP (H_2_O_2_/Fe^2+^, O_3_/UV, UV/H_2_O_2_, O_3_/H_2_O_2,_ O_3_/H_2_O_2_/UV…) [70].

AOPs are regarded as tertiary treatments performed at nearly ambient conditions when severe water quality constraints are required. [71]. These processes are gaining growing interest, but they still produce persistent traces of toxic chemical species unless performed in the presence of effective catalysts and/or promoters and further optimized [27,72,73,74,75,76,77,78,79]. In this regard, activated AOPs such as Photo-Fenton process (H_2_O_2_/Fe^2+^/UV) and its iron-free UV/H_2_O_2_ variant, H_2_O_2_-assisted ozonation (O_3_/H_2_O_2_) and H_2_O_2_-assisted photo-oxidation (UV/H_2_O_2_), UV/H_2_O_2_-assisted ozonation (UV/O_3_/H_2_O_2_), Electrochemical oxidation, ultrasound-activated oxidation, Supercritical Water Oxidation (SCWO) [80,81] and AOP-based hybrid technologies targeting synergistic effects [70] have turned out to be even more effective processes.

The most convenient oxidative method for organic pollutant removal is undoubtedly ozonation using mobile large-scale ozone generators, despite the energy consumption required to generate ozone [58]. Like other AOPs, ozonation mainly acts by generating a non-selective hydroxyl radical (•OH) in basic media [82,83] and preponderantly molecular ozone in acidic ones [84]. These media are by far more frequent compared to their neutral to alkaline counterparts in nature due to the presence of transitional and heavy metal cations along with already carboxylated organic species originating from the partial oxidation of organic matter. Molecular ozone is often regarded as more advantageous than highly reactive hydroxyl radicals, whose very short lifetime is a major obstacle due to the low and slow ozone dissolution in water [85,86].

Ozonation is also safer and more convenient than the Fenton process which also generates •OH radicals and other reactive species at room temperature in the presence of an Fe^2+^ + H_2_O_2_ mixture [87]. The main shortcomings of Fenton-like processes consist o the need for (1) reagents; (2) post-process Fe^2+^ recuperation using chelating agents with water contamination risks; and (3) constant pH adjustments to prevent free Fe^2+^ cation loss [88,89]. In addition, ozonation in moderately acidic media, more specifically in the presence of aluminosilicates, is probably the closest process to those occurring in soils and aquatic media containing clay suspensions, given the similarity in the distribution of most oxidized intermediates and end chain oxides, but are much more accelerated [18,19,20,24]. So far, some attempts to carry out the aluminosilicate-catalyzed ozonation of some organic pollutants allowed for total mineralization without traces of short-chain derivatives [2,59,84,90].

## 5. Ozone-Based Remediation Processes

Ozone exhibits an intrinsically high oxidizing power and polarity due to its various resonance states [91]. In water, ozone may act both directly via its molecular form and/or indirectly through its radical derivatives such as hydroxyls in alkaline media. The direct/indirect ozonation ratio depends on many factors, among which the pH and chemical composition of the water play significant roles [2,4,90]. Ozone acts via a direct attack on multiple bonds and phenolic groups of organic molecules [60,92], resulting in ketones, aldehydes and carboxylic acids [93,94,95], which completely disappear upon prolonged exposure to ozone and under specific operating conditions [96,97].

As a dipole, molecular ozone may also behave as an electrophilic and/or nucleophilic agent that promotes the formation of primary ozonide via 1–3 dipolar cyclo-addition to unsaturated bonds. The ozonide, in turn, is reactive towards ozone, and readily decomposes into carbonyl-functionalized compounds. This often occurs on aromatic and heterocyclic moieties, resulting in ring cleavage into C=O-bearing aliphatic derivatives or ring oxidation via a radical pathway. When acting as an electrophilic agent, ozone attacks take place on species or moieties bearing a high electron density such as deprotonated dissociating species like amines and phenols [98]. Conversely, the nucleophilic behavior of ozone involves attacks on electron-donating groups such as alkyl groups. During such a complex process, nitrogen and sulfur atoms, if any, are unavoidably oxidized. The hydroxyl radical is known to be much less selective than molecular ozone, and is produced in media containing hydroxyl anions [99]. Most polluted waters that need to be treated are fairly acidic because of the unavoidable partial oxidation of the organic pollutants, meaning that the action of molecular ozone prevails [100].

These properties confer an increased capacity for the adsorption of ozone onto solid surfaces and an increased capacity for the thorough mineralization of any organic compounds. This often requires a sufficient process duration according to their molecular structure and interactions with the host-polluted matrices in almost the same manner as this occurs in natural media [18,19,20,24]. Ozone was found to be even more effective than other oxidizing agents in organic pollutant removal without requiring reagents or bulky devices [2,101]. Ozone is more than ten times more soluble in aqueous media than compared molecular oxygen [102]. In different pathways according to the pH of the aqueous media, any organic impurity in waters behaves as ozone scavenger by readily reacting with ozone [103,104,105,106,107]. That is why ozone was first employed for drinking water production, disinfection or remediation through eco-friendly processes producing clean air or water devoid of any traces of organic substances or pathogenic bacteria [102,108,109,110,111]. Already oxygenated or chlorinated organic intermediates require even more pronounced ozonation due to their low oxidation capacity [2,90,92].

So far, a series of ozone-based methods have been tested for the decomposition of various categories of organic pollutants including phthalates, organic dyes, drugs and others. The data summarized in Table 2 provide an overview of the diverse types of solid catalysts used for this purpose and show that the catalyst’s effectiveness and thereby the removal yield of the organic substrate strongly depend on the operating conditions, more particularly the reaction time, the ozone/catalyst and ozone/substrate ratios.

Metal oxides such as MnO_2_ [156] and titania (TiO_2_) [157,158,159,160], alumina-supported TiO_2_ [117,123,124,161] and TiO_2_ in combination with activated carbon [161] were found to show appreciable catalytic activity in oxidative processes. Metal oxides’ surface properties have already been found to play significant roles in catalytic ozonation processes [162,163]. FeOOH is particularly interesting as a catalyst in the ozonation of many organic pollutants without necessarily resulting in high removal rates [114,134,142,147,164,165,166,167,168,169]. The catalytic activity was mainly explained in terms of its higher capacity to generate and convert HO_2_^−^ ions into hydroxyl and superoxide ion radicals in the vicinity of the catalyst surface.

Total mineralization of organic molecules can be achieved through ozone synergy with other oxidizing species (hydrogen peroxide, ultraviolet radiation, catalysts and/or promoting agents) [60]. Ozonation may also be activated with ultrasounds or microwaves, metal oxides, clays minerals, zeolites, activated carbons and metal oxides as produced or after suitable modifications [84,170,171,172,173]. The issue related to the low ozone solubility in aqueous media has often been tackled by using solid surfaces that promote adsorption [2,59,60,84,90,102]. In this regard, the effective elimination of organic pollutants has already been reported in the presence of oxides or clay minerals in aqueous media at adequate pH levels [2,59,60,92,174,175,176,177,178,179]. Solid catalysts such as FeOOH [142,147], bare and Co_3_O_4_-modified alumina [131,133,135,150,163,180,181,182,183,184,185,186,187], ceria-loaded activated carbon [148,188,189] and titania [151,152,190,191,192,193] display catalytic activity mainly based on the key contributions of multiple surface interactions and adsorption.

## 6. Iron and Solid Catalysts’ Effects on Ozone Consumption

Unless achieved with already-dissolved ozone in water, ozonation with or without catalysts is, by itself, a heterogeneous processes even when using dissolved catalysts to improve efficiency and ultimately lead to the total mineralization of the organic compounds [194]. Even in the presence of dissolved cations such as Fe(II), Mn(II), Ni(II), Co(II), Cd(II), Cu(II), Ag(I), Cr(III) and Zn(II), ozonation cannot be regarded as homogeneous since this process is triggered by ozone bubbling with gas–liquid diffusion hindrance acting as a kinetic-controlling step [195]. In addition, pH fluctuations may generate metal hydroxides that contribute through additional liquid–solid interfaces.

Iron is particularly interesting not only for its catalytic efficiency, as reported in several publications (Table 2), but also for its large occurrence in most natural media and lower toxicity compared to the other metal cations investigated so far. Among iron forms, the Fe^2+^ cation is probably the most reactive, and can be chelated by trace amounts of oxalates into even more reactive species [2,90,196].

Short-chain acids, aldehydes and peroxides can also generate Fe^2+^ complexes [2,5,84,197,198]. That is why the addition of oxalic acid turned out to be effective in enhancing various oxidative processes [60,92,169,199,200,201]. The free Fe^2+^ cation displays a high catalytic activity in oxidation processes which is even higher in its solid-supported forms. For instance, total COD removal could be achieved through a relatively short ozonation of sulfamethoxazole within 20 min in the presence of Fe^2+^ montmorillonite [60]. This was explained in terms of a synergy between the pH, Fe^2+^ cations and the clay surface.

Supported metal cations and nanoparticles along with insoluble solid catalysts such as metal oxides turned out to be interesting alternatives to free metal cations. They serve to prevent the accumulation of water and promote the contribution of the adsorption or at least the concentration of ozone in the surface’s vicinity, particularly for addressing the issue of ozone low solubility [174,202,203]. This beneficial effect of solid catalysts is reflected by their lowest ozone consumption combined with highest substrate removal rates, as illustrated by the lowest-to-highest substrate molar ratios of 0.045 for Fe^o^ particles, MnO_2_, Al_2_O_3_, 0.098 for Mn–Co–Fe oxide, 0.99 for iron silicate/FeOOH and 0.29 for Fe_3_O_4_ /MWCNt and NiFe_2_O_4_. Other low O_3_/S ratios were also observed for Fe_2_(SO_4_)_3_, H_2_O_2_/Fe/U and H_2_O_2_/Fe/TiO_2_/UV in the ozonation of terephthalic acid (0.625) or for Fe^2+^/UV in that of ibuprofen (1.0), but the effect of the molecular structure and of the contribution of the UV radiation must also be considered.

Metal oxides such as alumina [120,141,146,150,164,181], MnO_2_ [136,141,204,205], TiO_2_ [115,117,123,124,151,152,158,160,161,191,192], FeOOH [114,142,147,164,165,166] and ZnO [206], metals like Cu, Pt, Co [207,208]) on solid supports such as TiO_2_, SiO_2_, CeO_2_, Al_2_O_3_, activated carbons (AC) [209,210], mixed metal oxides [132,140,147,149,155,211], clays and clay minerals [60,209,212], metal-modified zeolites and modified and carbon-based materials [114,116,127,130,139,148,153,154,213,214,215,216] have become conventional solid catalysts for AOPs. Among these, alumina is currently one of the most investigated catalysts after being first regarded as ineffective [217], particularly in the degradation of hydrocarbons [92,218]. A judicious approach towards investigating the effect of the structure of the solid catalyst resides in comparing their performances for a given organic substrate, as illustrated by the example of dimethyl-phthalate (DMP) (Table 2). It is worth emphasizing that catalyst effectiveness cannot be evaluated only through ozone consumption, due to the effects of the physicochemical properties of the surface and textural features of the particle bulk. For instance, Co_3_O_4_/Mesoporous carbon aerogels and Fe^0^-multi-walled carbon nanotubes are supposedly highly porous catalysts with, however, fairly opposite performances in ozonation processes.

The effects of iron in nature are completely different from those of aluminum, copper or other metals, being essential for the global carbon cycle, not only as a catalyst in the redox processes but also a micronutrient for many species, particularly in marine biogeochemical cycles [219]. Iron is paramount for the human body and animals, being the core of blood hemoglobin in the form of an Fe^2+^ cation complex. The latter results from a chelating effect of five or six nitrogen atoms in a protein chain depending on the lack or capture of an oxygen molecule. Oxygen is captured without Fe^2+^ oxidation thanks to a protecting sterical effect of the protein hexadentate chelating site, thereby compensating for the weak solubility of oxygen in the blood plasma. In addition, iron deficiency is a cause of many human health issues including anemia, while an overdose of iron may enhance risks of hepatitis, cancer, etc. [220]. In nature and in foods, iron occurs in optimum concentrations, but the use of iron in oxidative treatments of waters like in Fenton processes may increase the iron contents, thereby imposing additional steps for iron removal. In ozonation processes catalyzed by aluminosilicates and soil-inspired materials, the iron content is limited by the cation exchange capacity (CEC). The latter has values in the same order of magnitude as natural media such as soils and aqueous clay suspensions, allowing such processes to be regarded as completely safe and harmless.

## 7. Soil-Inspired Materials for Ecofriendly Remediation

**Scheme 4:**
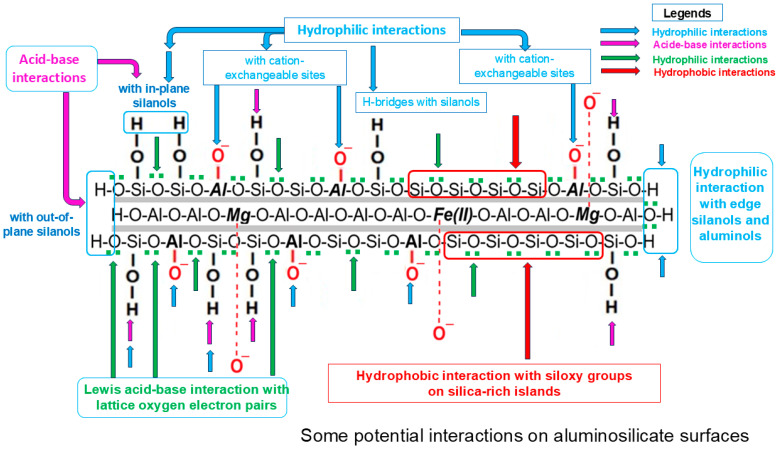
Some potential interactions on aluminosilicate surfaces.

Comparatively, soils are probably a less investigated media in terms of pollution prevention and the environmental remediation of organic pollutants. This is merely due to the instinctive approaches to tackling this issue being water pollution and post-pollution treatment and by assuming the efficacy of the non-demonstrated concept of self-regeneration in sufficient time when dealing with organic compounds. This last assumption has scarcely and barely been tackled through attempts to correlate the persistence of organic pollutants and their ecotoxicity to the presence and type of dispersed aluminosilicates in natural host media [18,19,20,24]. These attempts confirmed the key roles of not only soil self-regeneration using bacteria but also the adsorptive and catalytic properties of cationic clay minerals in oxidative processes, which in turn, appear to be governed by the types and distribution of the different interactions occurring between the pollutant and aluminosilicate surface (Scheme 4).

The clay fraction in soils and aqueous clay suspensions is probably the most important factor in organic pollutant capture and decomposition. Many works have demonstrated that the mere presence of clay minerals significantly improves the oxidative decomposition of a variety of organic molecules with respect to clay-free ozonation media and that adsorption has a marked contribution [2,20,59,60,84,92,175,176,177,178,221].

Organic pollutants adsorb onto clay minerals through various ways involving induced dipoles, electrostatic, hydrophobic–hydrophilic and/or Lewis acid–base interactions. Adsorption via electrostatic interactions (ion-exchange) takes place only for organic pollutants bearing exchangeable groups or moieties that acquire charges through protonation–deprotonation processes, which are pH-dependent.

No ozonation mechanisms have been established so far due to the difficulty of investigating the ozonation kinetics on solid surfaces, other than some attempts to provide a possible reaction scheme for ozone on a solid surface [2]. Scheme 4 shows that the adsorptive ability of aluminosilicates significantly contributes to their catalytic properties through the types and distribution of surface interactions with the organic pollutant.

Soils may promote not only oxidative processes at the interface with the atmosphere or oxygenated aquatic media, but also reductive transformations in deeper oxygen-poor layers. The presence of cations, more particularly Fe^2+^, was found to improve the catalytic activity of clay minerals such as montmorillonite or of SBA-like porous silica and others in the oxidative degradation of diverse organic pollutants such as drugs and organic dyes [24,59,60,84,92,176,178,179,221,222]. Most of these works and others [175,177,179] demonstrated the key role of the silica fraction in catalytic activity through its adsorptive properties and surface charge changes due to pH fluctuations. This finding is of great importance because it provides clear evidence that silica in soils must behave similarly and that silica-containing soils are capable of total self-regeneration within a reasonable amount of time in the case of slower contamination with such species. Consequently, they appear to be interesting catalytic media for the ecofriendly degradation of organic pollutants.

Pesticides can be degraded by different soil microorganisms [223,224,225,226,227]. Many fungi and bacteria are known to even mineralize some pesticides in soils [228], but care should be taken when dealing with partial decomposition since some derivatives can be more ecotoxic than the parent molecules. Furthermore, the incorporation of fertilizers, nutrients, soils conditions and others for soil amendment is expected to alter the soil’s capacity for the adsorption, migration and biodecomposition of pesticides. This is why a deep knowledge of soil’s physical and chemical behavior and more particularly that of the aluminosilicate fraction is an essential requirement to design an effective technique that allows for achieving the total natural elimination of the organic pollutant both in wet soils and in aqueous suspensions.

Organic air pollutants have a weaker impact on soils due to their lower concentrations compared to dissolved or dispersed organic molecules. The latter can significantly affect the normal physico-chemistry of soils and negatively impact biodiversity with direct and/or indirect effects on human health. Soils are directly polluted by the spread of pesticides and contact with contaminated wastewater or aquatic media. Consequently, soil pollution prevention and remediation are closely related to the impregnating liquids, particularly water.

## 8. Aluminosilicate-Based Catalysts

Porous crystalline aluminosilicates include cationic clay minerals with expandable 2D layered structures and zeolites with 3-D rigid scaffolds of SiO_4_ and AlO_4_^-^ tetrahedra connected by oxygen atoms [229]. Unlike clay minerals whose porosity varies according to the size of the entrapped species and hydration levels even up to total exfoliation and delamination, the zeolites frameworks display constant microporosity with a pore aperture close to the molecular size of monoaromatic hydrocarbons [2]. Such a porosity allows the diffusion of only small molecules such as monoaromatic hydrocarbons and their functionalized counterparts, e.g., phenol and p-benzoquinone [60,230]. So far, a wide variety of porous silicas, metal silicates and aluminosilicates including clay minerals, zeolites and their modified counterparts have been investigated in ozonation processes targeting organic pollutants elimination from waters; some of these are illustrated in Table 3.

However, both types of aluminosilicates display similar surface acid–base properties, a hydrophilic character on negatively charged surface and a hydrophobic behavior on silica-rich islands that make them suitable adsorbents for organic pollutants in wastewater [2]. The density of their surface charges and subsequently their silica-to-alumina ratio (SiO_2_/Al_2_O_3_) determine their cation-exchange capacity (CEC), acid–base properties, hydrophilic–hydrophobic tendencies and adsorption capacity [184,231,232]. Low SiO_2_/Al_2_O_3_ ratios promote a hydrophilic character and an affinity towards polar molecules. High SiO_2_/Al_2_O_3_ ratios favor hydrophobicity instead, with an increased affinity towards non-functionalized organic molecules [232]. This was found to have a beneficial effect on the ozonation of organic pollutants [92,175,177,179]. In addition, silica-rich zeolite ZSM 5 was already reported to show an even higher hydrophobic character and catalytic activity in the oxidation of small size molecules [127,230,233,234,235,236,237,238,239] compared to other zeolites such as H-Beta, H-Mordenite and H-USY [240].

**Table 3:** Some examples of organic pollutant ozonation catalyzed by silicas and aluminosilicates.

Substrate	Catalysts/Co-Oxidants	Removal (%)/Time (min)	Ref.
Methylene-blue, methyl-green, methyl-orange, methyl-thymol-blue	Acid-activated bentonite Na^+^-montmorillonite Fe^2+^-montmorillonite	100/5	[178]
Methylene-blue	Montmorillonite type K10	99/40 s (not total mineralization)	[221]
Malachite-green		95/40 s	
17α-ethynylestradiol	Fe^2+^-montmorillonite	96/1	[176]
17α-ethynylestradiol	SBA-15, SBA-16, MCM-41 MCM-48 mesoporous silica	100	[177]
Sulfamethoxazole, phenol	Fe^2+^-montmorillonite	100/10	[60]
Orange-G	Hematite/SBA-16	100/5	[92]
Diazinon	Na^+^-montmorillonite	100/30	[24]
Diclofenac sodium		100/10	
Bisphenol A	Acid-activated bentonite	90/30	[20]
Phenol	(Al-Fe)-pillared clay	100/25	[241]
Xylene	Cu/Al-pillared clay	28.2/60	[242]
Toluene		26.5/60	
Nitrobenzene	MnO_x_-/MCM-41 silica	88.9/10	[243]
Dimethyl phthalate	Ce/SBA-15; SBA-15	88.7/60	[122]
	Fe^o^/SBA-15-like silica	90–100	[125]
	Zeolite ZSM-5	>98	[127]
Remazol Black 5	Iron–Silica	70 (TOC) ^a^/90	[167]
p-Chloronitrobenzene	Iron silicate/FeOOH	99.8/15	[102]
p-Chlorobenzoic acid	Fe_2_O_3_/MCM-41-like silica	91.7/5; 88.6 (TOC) ^a^/60	[244]
Oxalic Acid	Co^2+^-montmorillonite	100/15	[59]
	Fe^2+^-montmorillonite		
	Fe^o^-SBA-15-like silica	86.6/60	[245]

^a^ TOC: total organic carbon measurements.

In addition, silica islands on aluminosilicates exhibit BrØnsted acidity through their silanol groups that undergo deprotonation at pHs higher than 5.6 [246] and Lewis basicity via the free electron pairs of lattice oxygen atoms in their siloxy groups (≡O_3_Si-O-SiO_3_≡). Both types of acidity of aluminosilicates seem to contribute to ozone adsorption and decomposition in water [233,247].

In the presence of alumina, silica and their combination as aluminosilicates, the pH is a key factor that determines the adsorption of the reactant, the catalytic properties and the desorption of the intermediates/products. On aluminosilicates, silanol’s behavior is strongly dependent on the pH of the impregnating media in the same manner as this occurs in natural clay-containing media. In ozonation processes catalyzed by alumina or zeolites, the pH of a solution even appears to determine the ozone consumption [248]. This is due to the fact that ozone acts differently at different pH values, i.e., direct and predominant attacks on molecular ozone under acidic conditions (pH < 4) and mainly via hydroxyl radicals beyond neutral pH [104,249]. Progressive ozone dissolution and pH fluctuations during ozonation are expected to strongly influence the catalyst activity and its interactions in aqueous media by modifying the prevalence of a specific action of ozone at the expense of another one [2,59,60,84,131,146,179,250]. In addition, the pH level should also influence most of the surface interactions of aluminosilicates in aqueous media including cation exchange. These interactions are known to be responsible for ozone adsorption on the surface and for the catalytic oxidation of the organic pollutants [233,251]. The hydration grade of zeolite seems to modulate the mobility of the exchangeable cation [252,253]. This cation mobility appears to be an essential requirement for effective oxidation of organic pollutants, as has already been reported for Fe^2+-^exchanged aluminosilicates [59,60,84,92,209].

When released on soils or in surface wastewater, organic pollutants are rapidly exposed to oxygen, sunlight radiation and oxidizing species including microorganisms. Previous works already demonstrated that aluminosilicates and, more specifically, clay minerals behave as soils particles and aqueous clay suspensions that enhance the oxidizing processes with almost similar intermediates and end chain products as those produced in natural media [18,19,20,24]. However, the use of microorganisms in remediation has always been subject to controversy due to the need for a large surface and a large amount of water, making it to difficult control process parameters and leading to an unavoidable impact on the environment. Among many attempts, except a few micro-organisms such as mixed microflora [226], fungal strains [227], *B. thiooxidans* and *S. paucimobilis* [254], the elimination rate of some pesticides scarcely reached 50–75% after 3–20 days [224,255,256,257]. A judicious design of green technology for ecofriendly remediation should adapt the most adequate oxidizing agents to the host media (microorganisms in natural media or ozone in water treatment facilities) and correlate the type of aluminosilicate to be used as the catalyst with the pollutant type and concentration and pH of the wastewater to be treated [2].

## 9. Conclusions

In conclusion, it appears that one of the major requirements for the implementation of the so-called concept of sustainable development resides in designing zero-waste technologies with no irreversible impact on the environment. This includes the issue of persistent organic pollutants. Given the failure of most oxidative treatments in achieving total mineralization of organic pollutants within a reasonable processes time and with a reasonable energy consumption, a new strategy is now arising through a trend to design green technologies that mimic the degradation processes occurring in nature. The use of ozone and clay minerals recently turned out to be a judicious combination for achieving eco-friendly oxidative processes with total depletion of all organic intermediates and trace amounts of harmless oxides. This was justified by the fact that ozone produces mostly similar intermediates and end-chain products regardless of the organic molecules as natural oxidative process occurring in clay-rich soils and aqueous clay suspensions. This can only be achieved through deep knowledge of the clay catalysts’ structures and surface interactions in correlation with the organic pollutant’s behavior in aqueous media. Advancing knowledge in this regard led to the conclusion that any aluminosilicate can display adequate acid-base properties and hydrophilic character at specific pH levels. These two conditions allow for the promotion of optimum adsorptive and catalytic properties for the capture and total degradation of a given organic molecule and its derivatives at a specific minimum time. Despite the fast and total disappearance of most organic pollutants in less than an hour, the lack of total mineralization is the cause of the persistence of intermediates with an unavoidable negative impact on biodiversity.

In addition, the presence of ion-exchangeable sites favors cation mobility in the vicinity of a solid surface, which turned out to be an essential requirement for achieving effective ozonation. Silica-rich islands on an aluminosilicate surface exhibit a beneficial organophilic character and available electron pairs on the lattice oxygen that promote, respectively, hydrophobic and Lewis acid–base interactions with organic molecules. Silanol can protonate or deprotonate, inducing variable negative charges that control the aggregation or dispersion of the solid particles and subsequently the extent of the adsorption surface according to the pH in the liquid media. The latter is also expected to determine the acid–base properties of functionalized organic molecules and their adsorption via electrostatic interactions according their pKa. Therefore, the aluminosilicate particles in soils and aqueous suspensions should display a variety of interactions, whose distribution can improve their adsorption and/or catalytic features in the oxidative degradation according to the targeted organic pollutant. This appears to be a common behavior of zeolites and clay materials, the latter offering additional advantages related to their natural availability and expandable structure that allow the diffusion and adsorption of a wider variety of organic substrates.

This work contributes to advancing knowledge on the behavior of organic pollutants in clay-containing media. The acid-base properties of aluminosilicates surface play a key role in their dispersion in aqueous/wet media and their catalytic activity in the oxidative degradation of organic pollutants. A deep understanding of the aluminosilicate–organic substrate interaction allows for their persistence to be predicted according to their molecular structure, contact time and the type of clay-containing media.

The ozone/clay catalyst combination appears to be a judicious nature-inspired approach that simulates accelerated natural oxidative degradation processes for organic molecules. This process could even lead to total remediation, when the kinetics of these natural processes are taken into account and if the amount of catalyst and pH are correlated to the amount and molecular structure of the organic substrate. Knowledge of the influences of the clay structure and composition provide valuation information for understanding the self-regeneration capacity of clay-containing natural media. The wide variety of organic pollutants could partly justify the lack of clear strategies in green industrial technology sharing common wastewater treatment facilities downstream. Economic reasons excluding any environmental considerations and long-term sustainable development are the main obstacles in this regard. Clay-catalyzed ozonation still remains a viable technique in at least some steps in water treatments notwithstanding that the implementation of ozonation-based processes for water treatment is still facing major obstacles. The latter reside mainly in a relatively high energy consumption for ozone production, corrosivity for metals and unavoidable oxidation of bromide into bromate, more particularly in drinking water. Bromate appears to be a potential source of cancer for humans according to some tests on rats. The issues related to hypothetical complexity of ozonation devises/ozone generator and ozone toxicity seem to be less problematic than those induced by chlorine and other oxidizing agents including Fenton reagents.

## Figures and Tables

**Table 1:** Physico-chemical features, toxicity and elimination rate of some common pesticides.

Organic Species	Chemical Formula	Solubility (mg L^−1^)	Toxicity	Removal Method	Elim. Rate (%) ^c^	Ref.
α-Endosulfan	C_9_H_6_Cl_6_O_3_S	0.51	0.52 ^a^	Biodegradation in soils	>90	[6,7,8,9,10,11]
β-Endosulfan		0.45				
Atrazine	C_8_H_14_N_5_Cl	30–33	0.128–40 ^a^	Adsorption/activated carbon *	75–83	[6,12,13,14,15,16,17]
				Adsorption/organo-montmorillonite *	93–96	
				Nanofiltration/membrane *	95	
					85	
				Clay-catalyzed ozonation at moderately acidic pH	100	[18,19,20]
				Tourmaline-catalyzed ozonation	98	[21]
Bupirimate	C_13_H_24_N_4_O_3_S	22	1–50 ^b^	Wastewater electrochemical treatment	74 (COD) ^d^	[22]
Carbaryl	C_12_Hl_1_NO_2_	<1%	23.9/4 days ^a^	-	-	[6,23]
Diazinon	C_12_H_21_N_2_O_3_PS	40.0	105	Clay/ozonation in water at moderately acidic pH	100	[18,19,24]
Dichlorodiphenyltrichloroethane (DDT)	C_14_H_9_C_l5_	0.1	41.5 ^a^			[25,26]
Diuron	C_9_H_10_Cl_2_N_2_O	42	28.3 ^a^			[27,28,29]
Endrin	C_12_H_8_Cl_6_O	0.00025	-	Photo-Fenton *	-	[30]
Etridiazole	C_5_H5Cl_3_N_2_OS	117	>945 ^b^	Photo-degradation *	90	[31]
Fenitrothion	C_9_H_12_NO_5_PS	21–38	-	Fenton/wastewaters	>90	[32]
Flutriafol	C_16_H_13_F_2_N_3_O	130	310–67,000 ppb	Wastewater electrochemical treatment	85	[33]
Glyphosate	C_3_H_8_NO_5_P	12,000	6.62 ^a^	-	-	[27,34,35]
Lindane	C_6_H_6_Cl_6_	7.9–8.4	400 ^a^ 710 (cell) ^a^	Fenton *	>90	[30,36,37]
Parathion	C_10_H_14_NO_5_PS	20	0.9–10 ^b^	Wastewater ozonation	>90	[6,38,39,40,41]
Methyl-Parathion	C_8_H_10_NO_5_PS	55	10–67 ^b^		>90	
Diazinon	C_12_H_21_N_2_O_3_PS	40.0	105		100	
Malathion	C_10_H_19_O_6_PS_2_	145	0.085/4 h ^b^	Adsorption/resin amberlyst-15	96	[42]
Lindane	C_6_H_6_Cl_6_	7.9–8.4	400 ^a^ 710 (cell) ^a^	Fenton *	>90	[30,36,37]

^a^ Half maximal effective concentration (EC_50_) as median concentration expressed in μg L^−1^. ^b^ Lethal dose: LD_50_ (median value), expressed in mg/kg for domestic and farm animals. ^c^ Elimination rate by adsorption or ozonation (%). This factor does not necessarily involve total mineralization. ^d^ COD: Chemical Oxygen Demand. * Removal from drinking water. The solubility values, expressed in mg L^−1^, were measured in distilled water at different temperatures in the range 20–25 °C. The toxicity ranges presented in this table include different values provided by different data sources for the same organic species.

**Table 2:** Examples of catalytic ozonation processes of some organic pollutants.

Substrate	Catalysts/Co-Oxidants	Operating Conditions *	Removal (%)/Time (min) **	O_3_/S ^a^	Ref.
Dibutyl phthalate	NiFe_2_O_4_	O_3_/catalyst mol/Wt ratio = 156.25	100/60	568.18	[112]
	Mn_0.95_BiFe_2_O_4_	-	69.6/60	0.001	[113]
	FeOOH/AC ^b^	[Catalyst] = 10–100 mg L^−1^	63/60	4.15	[114]
	TiO_2_ films/UV	-	46–85	183.82	[115]
	Activated carbon	-	94	-	[116]
Terephthalic acid	Fe_2_(SO_4_)_3_, H_2_O_2_/Fe/UV, H_2_O_2_/Fe/TiO_2_/UV	O_3_/catalyst mol/Wt ratio = 0.035–0.045	40–100/Depending on process	0.625	[117]
Acid Red 18	Ca(OH)_2_	-	100 (TOC) ^c^/60	1.82	[117]
Bis(2-ethylhexyl) phthalate	Mn^2+^, Co^2+^, Fe^2+^, Cr^3+^	O_3_/catalyst mol/Wt ratio = 0.26 × 10^−4^	45–75/Depending on catalyst	-	[118]
	Activated carbon	-	94	-	[116]
DiMe/DiEt phthalates	Activated carbon	-	94	-	[116]
Dimethyl- phthalate	Fe_3_O_4_ SiO_2_-Fe_3_O_4_ CeO_2_/SiO_2_-Fe_3_O_4_ La_2_O_3_/SiO_2_-Fe_3_O_4_ Pr_6_O_11_/SiO_2_-Fe_3_O_4_	[Catalyst] = 0.5 g·L^−1^	96.4 Fe_3_O_4_ 94.8 97.6 98.1 98.4	13.88	[119]
	γ-Al_2_O_3_	-	100/25; 46 (TOC) ^c^; 75 (COD) ^d^	125	[120]
	RuO_2_/Al_2_O_3_/microwave	[Catalyst] = 10 g·L^−1^	100%	-	[121]
	Ce/SBA-15	-	88.7/60	6250	[122]
	UV/TiO_2_	-	89.9 (TOC) ^c^	4688	[123]
	UV/TiO_2_-γ-Al_2_O_3_	-	90–100	0.005	[124]
	Fe/SBA-15	-	90–100	52	[125]
	Ru-Al_2_O_3_/O_3_	-	100%	172.14	[126]
	O_3_/UV/O_3_/zeolite ZSM-5	[Catalyst] = 4 g·L^−1^	>98%	0.25	[127]
Reactive Blue 19	Co_3_O_4_/Carbon aerogel		99/5	0.054	[128]
Methylene Blue	Fe^0^- MWCNt ^e^	O_3_/catalyst mol/Wt ratio = 434	89 (TOC) ^c^ /125	4167	[129]
Acid Orange II	MgFe_2_O_4_	O_3_/catalyst mol/Wt ratio = 0.084	94.1/40; 48.1 (TOC) ^c^ /160	3.0	[113]
Orange II	Mo		100/20;	-	[114]
Basic Blue 3	Plasma treated magnetites	O_3_/catalyst mol/Wt ratio = 0.3	93.47/15	3.12	[115]
Basic Violet 3	Fe/AC ^b^	O_3_/catalyst mol/Wt ratio = 0.062	>96/30; 57 (COD) ^d^ /90	2.57	[130]
Reactive Black 5	Pyrite cinder (PyC)	-	17.39	-	[131]
	Ce-PyC	-	42.12		
Acid Red B	Fe–Cu	O_3_/catalyst mol/Wt ratio = 0.15	90/20; 70 (COD) ^d^ /60	2.08	[132]
Remazol Black 5	Iron–Silica	O_3_/catalyst mol/Wt ratio = 0.23	70 (TOC) ^c^ /90	4.16	[133]
p-Chloro-nitrobenzene	Iron silicate/FeOOH	-	99.8/15	0.99	[134]
	MnO_2_-based catalysts	-	51.7–68.0	0.032	[117]
	Zn-Cu/silicate-polymer	-	99.3/15; 51.2 (TOC) ^c^ /15	2 × 10^−6^	[135]
p-Chlorobenzoic acid	Fe_3_O_4_-Al_2_O_3_	-	100/30	233.75	[113]
	Fe_2_O_3_/Zeolite MCM-41	O_3_/catalyst mol/Wt ratio = 1.76	91.7/5; 88.6 (TOC) ^c^ /60	165.23	[114]
Sulfosalycilic acid	CeO_2_/MnO_2_	-	100 (TOC) ^c^ /30	0.44	[136]
Thymol	Fe_3_O_4_-SiO_2_-Yb_2_O_3_	-	92/30	44.78	[137]
Ethylbenzene	FeSO_4_	[O_3_]_0_ = 25 mg L^−1^ [Catalyst] = 0.08 mmol L^−1^	60/60	39.1 × 10^−4^	[138]
chlorobenzene,			19.9/60		
1-Cl-2-methyl2-butene			90/60		
Naphthalene			85.3/60		
Phenol	Fe_3_O_4_ /AC ^b^	-	98.5/60; 69.8 (COD) ^d^ /240	7.75	[139]
p-Nitrophenol	Mn–Co–Fe oxide	-	95 (TOC) ^c^ /60	0.098	[140]
	Fe^o^, MnO_2_, Al_2_O_3_	-	89.5 (COD) ^d^ /60	0.045	[141]
Oxalic Acid	Fe^o^-SBA-15-like silica	O_3_/catalyst mol/Wt ratio = 60.7	86.6/60	93.98	[115]
	FeOOH	-	54/30	28.0	[116]
Sulfamethazine	Ce_0.1_Fe_0.9_OOH	-	42.1%	0.075	[142]
Phenacetin	CuFe_2_O_4_	-	95/30 min	0.00018	[143]
Acetaminophen	MgO	-	94%/30 min	0.0009	[144]
Ibuprofen	Fe^2+^/UV	Wavelength 300 nm [Catalyst] = 0.25 mM	97/10	1.0	[145]
	Al_2_O_3_	-	8.6–13/30	-	[146]
	β-FeOOH/Al_2_O_3_	-	100/9 min; 90 (TOC) ^c^ /40	-	[147]
Sulfamethoxazole	CeO_2_/Activated carbon,	-	58/180; 72.6 (TOC) ^c^	-	[148]
	CeO_2_/MWCNt ^e^	-	33/180; 55.9 (TOC) ^c^	-	
	Co^2+^-montmorillonite	Fully ion-exchanged Mt. Metal(II)–Mt [Catalyst] =: 1.91 g L^−1^ Concentration = 3 × 10^−4^ M; initial, pH 2.88	97 (COD) ^d^	0.21/0.30 ^f^	[60]
	Ni^2+^-montmorillonite		95 (COD) ^d^		
	Cu^2+^-montmorillonite		92 (COD) ^d^		
	Na^+^-montmorillonite		85 (COD) ^d^		
	Fe^2+^-montmorillonite		98–100 (COD) ^d^		
Phenazone + Diphenhydramine-HCl	Fe–Co/ZrO_2_	-	60% PZ/40; 40% DP	-	[149]
Ethynyl-estradiol + Sulfamethoxazole + diclofenac	Co_3_O_4_/Al_2_O_3_	-	100%/10	-	[150]
Diclofenac	TiO_2_/UV-A	-	100; 80 (TOC) ^c^	-	[151]
2-Ethylhexyl-trans-4-methoxycinnamate	TiO_2_	-	20/15	-	[152]
Oil refining wastewater	Fe(NO_3_)_3_/AC ^b^	pH = 7.8; [Catalyst] = 5 g·L^−1^ [O_3_]_0_= 10 g·L^−1^ Flow rate = 45 L·h^−1^	33.7 (COD) ^d^ /60	2.0	[153]
Omethoate	Fe(III)/AC ^b^	-	50/120	9.0	[154]
C_5_H_12_NO_4_PS					
p-Chloronitrobenzene	Iron silicate/FeOOH	-	99.8/15	0.99	[134]
Aniline	FeSO_4_	-	84/60	39.1 × 10^−4^	[138]
Organic matter	Mn-Fe-Cu/Al_2_O_3_	-	67.1 (COD) ^d^/60	378.16	[155]
	Fe(NO_3_)_3_/AC ^b^	-	33.7 (COD) ^d^ /60	607.09	[153]

^a^ S: substrate or organic molecule. O_3_/S molar: Ozone/substrate mole ratio. ^b^ AC: activated carbon. ^c^ TOC: total organic carbon measurements. ^d^ COD: Chemical Oxygen Demand measurements. ^e^ MWCNt: Multi Wallet Carbon Nano-Tubes. ^f^ O_3_/S molar/time: Ozone/substrate mole ratio per minute. * O_3_/catalyst Wt. ratio. ** Some data related to the removal rate (%)/time (min) are completed by additional value of the COD or TOC elimination rate in brackets.
